# Phrenology and Character

**Published:** 1863-04

**Authors:** Edwin Goadby


### Art. VII.—PHRENOLOGY AND CHARACTER.

By Edwin Goadby.

The history of phrenology is one of the most interesting departments of human opinion. As a science, if it be one, it directly or indirectly affects so many vital, social, and abstract questions as to challenge almost every intellect at some stage of its development, but as an exhibition of the fluctuations of human opinion it seems to possess something extraordinarily unique. Nowhere, perhaps, do we meet with so much modifying and re-classification. Faculties are invented like new designs in lace, and the supposed discoveries of the majority of its chief supporters prove to be rather alterations than originalities. Rarely has a sturdy opponent risen against it, but it has yielded to him first one thing and then another, a region in one place as unknown, and in another as miscalled, until it would seem that the whole scheme must be recommenced. Until very recently, the volume of the brain was regarded as a decisive test of its powers; but recent discoveries in relation to the varying composition of nerve-tissue and other modifying conditions, have entirely disproved it as such beyond the line of demarcation where the idiotic tendency is eliminated. The frontal sinuses have also been another point upon which there has necessarily been a qualification, if not a shifting of position. The cerebellum, too, has been a great battle-ground, and the weight of opinion and the testimony of facts are now so evenly balanced for and against its phrenological position, as must invalidate any logical claims assumed on either basis without further researches, notwithstanding that Spurzheim maintained it was "impossible to unite a greater number of proofs in demonstration of any natural truth, than may be presented to determine the function of the cerebellum." Many physiologists also complain that the brain has been cut off from the rest of the nervous system, and the cautious and searching criticisms of Mr. G. H. Lewes on the reflex-theory, which still farther involved matters, and his axiom that identity of nerve-structure everywhere implies identity of properties, with its important corollaries, threatens the citadel itself; and when once the spinal cord is viewed under the new relations his own and other researches suggest, if they do not establish, an entire change will be necessary in the major part of its classifications.

Later still, matters do not seem to improve. The discussion of the claims of phrenology by Professor Bain, of Aberdeen,* discloses still further uncertainties, notwithstanding that it has the singular merit of being the production of one who leans strongly upon phrenology, even whilst he unsparingly reveals its deficiencies and makes havoc with its classification. The propensities, the sentiments, and the intellectual faculties are all minutely and successively examined, and their confusion and incoherence

---

* *On the Study of Character, including an Estimate of Phrenology.* By Alexander Bain, A.M., Professor of Logic in the University of Aberdeen. London: Parker, Son, and Bourn.

strikingly exhibited. A separate chapter is also devoted to the omissions of phrenology, and the general impression his book leaves upon the mind is that of the exceedingly crude and unscientific character of phrenology, even when viewed by him at its best as a science of character and not a science of mind.

To us, however, whatever would invalidate the older claims of phrenology as a science of mind, must necessarily affect her new ones as a science of character; and after having followed the Professor in his criticisms this result is very apparent to every one but the critic himself, who nowhere delivers a deliberate opinion either one way or the other, except to show that there is no certainty either way. The best thinkers, indeed, of the present day, who regard a science of character as not only possible, but in one of its hopeful stages of formation, very plainly tell us that it can only be based upon a science of mind. It may be all very well to grow absurdly revolutionary, and complain that the only reason previous philosophers have failed in this respect is because they have endeavoured to unmask the subtle laws of mind, and deduce special faculties by a simple analysis of consciousness when a purer inductive method were better; but it is only the shallow and the unthinking who can be so easily led away. History, when fairly examined, yields no such barren results. Perfection is not possible, except by a steady cumulative growth. Philosophical systems may have failed, but they served their purpose, like scientific ones, and only failed from the pride of their authors, who would impose their formulas upon humanity like restrictive chains, and forget that the world has a future, and many geniuses are yet unborn—an error from which even phrenology is not free, since it aims to do precisely the same thing by a chemical decomposition of the mind into certain organic primaries. Its followers taunt the metaphysicians with the perplexities in which their method has involved them, and smilingly, yet caustically, assure them that they are easily obviated by an ultimate analysis, and an accurate organology such as they have the honour to institute and propound. There can be no unfairness, therefore, in applying to phrenology its own test of other systems, and immediately we do so we must write failure over its reiterated pretensions and most sanguine expectations. Its own classification proves to be as unsatisfactory as any metaphysical one, and its history is as full of changes and confusions as any one of the vexed questions of the older science, and the more so that it hitherto occupies so insignificant a period of time as compared with the centuries of which so much is made on the other side. As a science, nothing could be at first simpler than phrenology, and yet to what complexities has it not grown, scattering along its track continued evidences of its hastiness, incoherence, and disorganization? This, indeed, any candid phrenologist must at once admit, if pressed, even when he may not forego the usual complaint against metaphysical studies.

Metaphysicians have undoubtedly discovered certain very important mental laws, otherwise not arrived at, even when from their universal validity they were always in some way or other tacitly assumed by those most ignorant of them. An unreasoning disregard of these native forms and invariable modes was sure to lead into error. Perhaps the one party were wrong in endeavouring to bring every faculty into consciousness without a due regard for differences of temperament and race; but were the other therefore justified in putting consciousness entirely out of court, in isolating every faculty, and in regarding all mental laws to have their origin in physical ones? The classification of the second party might be perfect, but still every organ must have its law of connexion with every other, and how was that to be demonstrated? It could not be done upon physiological grounds, for there all distinctions immediately ceased, in so far as cognisable by our senses; and having already declared all others to be useless, what were they to do? This is a question intimately connected with the science from its very formation, and yet one which, as far as our knowledge extends, no phrenologist has ever answered. Nevertheless, we think they are bound to answer it. Their organology still remains without any synergy being demonstrated, and Abernethy's comparison of the mind, under their view of it, to "a committee of organs, with a general board," would be sufficiently exact, if they had indicated the locality of the board-room, and the precise system of communication. As it is, all phrenology has done for mental science, irrespective of its many physiological impulsions, is to make out of the brain a sort of blind comparison to the key-board of an instrument, moved upon by an unknown mysterious Will, or Me, whether originating in the brain, or without it, it cannot say, and about which believers tell us they know nothing, nor does any one else. So that even when accepted as a scientific ultimatum, we are necessarily thrown back by it upon some other studies of mind; and its very elimination at starting of what it regarded as the source of all previous perplexities—namely, a search for native forms and laws by an examination of the mind's own operations, has still to be attempted before its own system can have any rational unity. If this be not a curiosity in scientific circuition, we do not know what is one.

Such a want of unity could not possibly escape any acute observer, and hence M. Comte, in discussing its many imperfections, thus calls attention to it:—

"The two laws of action,—intermission and association,—

require much more attention than they have yet received in connexion with cerebral physiology. The law of intermittence is eminently applicable to the functions of the brain, the symmetry of the organs being borne in mind. *But this great subject requires a new examination,* seeing that it is requisite for science to reconcile their evident intermittence with the perfect continuity that seems to be involved in the connexion which mutually unites all our intellectual operations, from earliest infancy to extreme decrepitude, and which cannot be interrupted by the deepest cerebral perturbations, provided they are transient. This question, for which metaphysical theories allowed no place, *certainly offers serious difficulties:* but its positive solution must throw great light upon the general course of intellectual acts. As for the association of the faculties, in sympathy or synergy, the physiologists begin to understand its high importance, though its general laws have not yet been scientifically studied. Without this consideration, the number of propensities, sentiments, or aptitudes would seem to be susceptible of any degree of multiplication."*

It is scarcely needful to say, by way of qualification to the assumed futility of metaphysical theories, even in reference to these mental phenomena, that the investigations of Sir William Hamilton upon consciousness, and his theory of latent modifications, have done very much to answer the problem propounded.

Not only does phrenology break up what Mr. Bain calls "the great fact of our spontaneous energy, which lies at the basis of will, and determines the strength or weakness of our active impulses generally,"† but its very analysis is neither correct nor ultimate. It frequently comprehends, under distinct organs, what is really but one and the same aspect of mind when carefully examined. Thus, each of the two organs called adhesiveness and benevolence, concentrativeness and firmness, combativeness and destructiveness, are shown by Mr. Bain to have the same root. Under self-esteem and conscientiousness we have really a plurality of ideas which a very little precise thinking will serve to clear up, and the boundary lines between the former and love of approbation are exceedingly indistinct. In the sentiment of hope, a purely secondary faculty, determined, on their own showing, by general constitution and temperament, is put for a primary one, and is erected into a weak antithesis to cautiousness, which still farther misleads. Cautiousness itself is pre-eminently a confusion, and a fatal one, for it includes fear, circumspection, and foresight. Now fear, as Mr. Samuel

---

* *Positive Philosophy.* Translated by Harriet Martineau, vol. i. pp. 477-8.

† *Study of Character, &c.*, p. 117.

Bailey has aptly remarked, is an emotion, but foresight is an intellectual act, even when attended by, or resulting from an emotion. On a consideration of the organ and the faculty in this light, both fall together. The same writer has cleverly illustrated this by an apt example. "It is well known," he says, "that the Duke of Wellington, whose courage was unquestionable, and who was certainly not subject beyond his fellow-soldiers to needless or easily-excited alarm, was one of the most circumspect generals that ever conducted a campaign or fought a battle; and his foresight reached to the minutest as well as the most comprehensive arrangements needful to carry out his purpose. In respect of these latter qualities, he ought to have had the organ *large;* in respect of fear, he ought to have had it *small.*"* The confusion here is perhaps more marked, but is certainly not greater than that exhibited under the faculty of ideality, as interpreted by Combe. We must quote Mr. Bailey again. "Mark the number of things which a single faculty or organ is here represented as doing; it produces feelings, and itself experiences delight; it also desires what is preternaturally requisite, as well as rejoices; further, it endows all ideas with splendid excellence; it stimulates other faculties to exercise their imaginations; it inspires with exaggeration and enthusiasm, and it prompts to embellishments and brilliant conceptions. In this crowd of operations, real and fictitious, huddled together without congruity, you seek in vain for any principle of classification."†

Nor is this all; for this faculty is already assumed in conjunction with the highest exercise of every other, the truth being, not only, as Mr. Bain adds, that "every one of the phrenological sentiments leads to the formation of ideals; wonder large makes a man invent marvels, and benevolence may operate solely in an ideal direction," but that, as every faculty is stimulated "to imagine scenes and objects," to use Combe's words, there is what we cannot call less than ideality in a greater, smaller, ruder, or more refined degree in every propensity, sentiment, and intellectual faculty; and were our powers of penetration keener than they are, we should discover this under guises where we least expect it. Even hunters, farmers, grooms, and butchers have a large share of this simpler kind of ideality, which takes a vigorous form from a certain dumb fascination animals and external nature have over them, the only difference between their poetry and that of the singer or the student being expressed, according to Emerson, "in their choice of life, and not in their choice of words."‡

* *Letters on the Human Mind*, quoted by Bain, p. 103.

† Ibid., p. 135. ‡ *Essays*, second series, "The Poet," p. 10.

These confusions grow upon us as we proceed. We come now to a part where physiology should have helped them to have avoided error, even when it led them to no definite truth. The researches of Dr. Brown, Sir William Hamilton, Sir Charles Bell, Professor Bain, and others, tell with terrible effect upon six of the supposed intellectual faculties. Thus form, size, weight, order, eventuality, and time, receive an entirely new exposition by the revived notion of a muscular sense, which, however, until lately, had never received anything like justice at the hands of physiologists and psychologists, although Sir William Hamilton, in his *Notes to Reid*, shows that Aristotle himself calls "Motion and Rest, Magnitude, Figure, and Number," the common concomitants of sight and touch; and the schoolmen added to them Place, Distance, Position, and Continuity.*

We have not space to detail these criticisms, but they will be found at some length in the writings of the authors already referred to, and will amply repay a careful study. Constructiveness, amongst the propensities, evidently falls into the same group, being dependent upon peculiarities of nervous and muscular organization, combined with a discriminating power regulated by the eye, neither of them requiring any distinct cerebral convolution or organ. Colour, too, is optical sensibility, which, however, may sometimes amount to a distinct gift, and is very characteristic of all tropical races, whether their organology would indicate it or not. Locality is defined by Mr. Bain as a combination of form and colour, stimulated by a purely local interest. It is by no means certain how far Language can be considered as a distinct faculty, inasmuch as so many subsidiary facts are grouped together under the term, as memory, vocal readiness, the result of constitutional vivacity and a happy adjustment of cerebral and muscular power, accurate conceptions, fancy, and a discrimination in the choice of expressive phraseology. Again, Individuality, Comparison, and Causality, seem to us to have little distinctness as separate faculties, and that only of the haziest kind, for all three seem to trace the ascension of what Kant has called the pure reason, which begins with analysis and proceeds to synthesis, each being, as Sir W. Hamilton says, the relative and the correlative of the other, and both constituting a single method in the research of causes.†

In sensation and emotion phrenology presents many deficiencies. There is no place for Taste, in which most individuals vary; no mode of estimating varieties of the sense of Smell; and Hearing is very inadequately represented under Tune.

---

* Reid's *Works*, p. 124, edit. 1852. † *Lectures*, vol. i. pp. 98, 99.

There is no such emotion as that of Pursuit or Plot-interest, no Love of Truth, and no Fine Art susceptibilities, other than those confusedly assumed under Ideality, so that the picturesque and the sublime have no lines of distinction. Prudence, too, can hardly be considered as given under Cautiousness. We miss any hints of politeness, of brusqueness, and of plain straightforwardness, or of adroitness in manœuvring, save as the latter may be given as a propensity under Secretiveness, whereas there is much intellect in it. What Mr. Bain calls "the intellectual element of disinterestedness, Sympathy," hovers very vaguely about Benevolence, whilst for any special gifts over and above these, such as genius confers, there is no other method than that of measurement by the preponderance of one organ over another, so that four lines difference in the height of an organ should be conclusive evidence of unusual power.

Then there is memory, which gets even more abused than imagination. Ideality shows the latter about as confusedly as is well possible; but what shall disclose the former? If no cerebral convolution can be found for it, or there be no necessity for allowing one, as is always tacitly assumed, then it follows that it is somehow bound up as an integral portion of the mind. The supposition then will startle one,—if so large a faculty be diffusive, wherein lies the argument for making others less so? Why give to Ideality a local seat, and make it at the same time a diffusive power? Why do the same for Individuality, Comparison, and Causality? By what line of argumentation can such a proceeding be justified? The whole of the emotions, too, are as diffusive as memory; and why locate them? The memory is, indeed, the oldest and the latest battle-ground of many anti-phrenologists, and no one will thank us for going further into the subject, although in the above questions there is, we think, opened a new line of attack from their own side, by which organologic aspects are rendered still more doubtful. That memory is a purely physical sense, a molecular tenacity merely, every genuine psychologist has always more than doubted, even when analogies like the persistence of the effect of vaccination helps to establish it as by no means a solitary wonder; but what it is beyond a power of reproduction having two terms of relation—those of simultaneity and affinity, which Sir W. Hamilton has carried up into a single higher law, styled the law of Redintegration or Totality, is no easy matter for any one to state. Mr. Bain's definition of it, as a general property of the mind, modified by local susceptibility, rather hands the matter over again to the phrenologists, notwithstanding that, apart from any theory, it is as explicit as one can expect.

The remarks of M. Comte in reference to this portion of our subject deserve attention, chiefly from the fact that the position he occupies in the matter is so entirely different to our own. He complains that the position of phrenology in this respect is as unsatisfactory as in any other, the enumeration of the various faculties being conceived of in a very superficial way, and there being no check to further and continued innovations.

"Unless a sound philosophy interposes to establish some order, we may have as many faculties and organs as the psychologists of old made entities. However great may be the diversities of animal natures, or even of human types, it is yet to be conceived (as real acts usually suppose the concurrence of several fundamental faculties) that even a greater multiplicity might be represented by a very small number of elementary functions of the two orders. If, for instance, the whole number were reduced to twelve or fifteen well-marked faculties, their combinations, binary, ternary, quaternary, &c., would doubtless correspond to many more types than can exist, even if we restrict ourselves to distinguishing, in relation to the normal degrees of activity of each function, two other degrees, one higher and the other lower. *But the exorbitant multiplication of faculties is not in itself so shocking as the levity of most of the pretended analyses which have regulated their distribution.* In the intellectual order especially, the aptitudes have been usually ill described, apart from the organs, as when a mathematical aptitude is assigned on grounds which would have justified our assigning a chemical aptitude or an anatomical aptitude, if the whole bony casket had not been previously parcelled off into irremovable compartments. If a man could do sums according to rules quickly and easily, he had the mathematical aptitude, according to those who do not suspect that mathematical speculations require any superiority of intellect. Though the analysis of the affective faculties, which are so much better marked, is less imperfect, there are several instances of needless multiplication in that department."*

To a radically defective ultimate analysis may also be added so imperfect a verification of their several organs, as not only throws discredit upon their organology itself, but indicates the very method itself to be erroneous through which they seek to establish it. In the first place, phrenology has to prove that it is an unexceptional law, that every convolution so communicates its form to the external cranial surface as to be readily recognisable without any possibility of error. As yet it has never done this, and can never be expected to do it, as those

* *Positive Philosophy*, vol. i. pp. 474, 5.

who have examined any one case of a careful post-mortem disclosure can readily testify—a convolution showing itself to be only strictly readable from the inner side of the skull. We are even then unable to discover how far any one important prominence is the effect of collateral pressure, since each may absolutely indicate a larger amount of power in other adjacent ones by no means so prominent to the view. This latter fact was sufficient to overturn the primitive form of a tier of organs when urged by Meiners, and Gall at once gave up to it the organs about the base and centre of the brain. But it even now presents a difficulty the most cautious observer can never be sure he has completely mastered. Secondly, there yet lacks scientific historical evidence to show that the characteristics of the cranial surface are altered after the age of puberty, or that certain faculties, after continued and exclusive use, have ever arisen in the least beyond their fellows. So simple a test as this, if fully and repeatedly demonstrated, and checked by a rigorous *experimentum crucis*, would show a believable ground whence the scepticism of many in the other departments might be easily and victoriously met. Thirdly, the evidence for the location of special organs has hitherto always commenced by a genuine guess, whatever its subsequent process may have been. Gall usually selected a person with a strong characteristic, and then sought a prominent cranial aspect as its organ, which, upon further evidence being supplied by other cases upon the same basis, was regarded as a scientific fact. Now, as he could never know, in the first place, how far the supposed organ was due to accidental circumstances of growth in infancy, and in the second place, how far its prominence was due to collateral causes, supposing for the moment that every convolution impressed itself accurately through the bony plate, he was never proceeding safely or scientifically by continuing the method of agreement unchecked by any other. The first uncertainties must necessarily vitiate all subsequent conclusions, and a single negative instance might let in disturbances which would tend to produce unlooked-for results. So that unless the same fact were established by other methods—that of difference, for instance, the same number of cases being shown not to have the organ who had not the faculty—the entire process must ever be open to doubt, and leave so large an opening for variations as in a larger average of thousands, or millions, instead of hundreds, might introduce quite a different law. The remarks Mr. Bain makes under amativeness have great force here. "Granted," he says, "that several hundred cases have been observed with all these precautions (referring to education, temperament, and the character of the parts especially involved, and not the tests we have mentioned) which may be fairly doubted, we still desiderate the continuance of the observations until they have embraced thousands of instances. Considering that the inference from them extends to the millions of the human family, we should like the actual verification to extend to a larger proportion than that at present of the actual individuals."*

Two instances are given by Sir William Hamilton, wherein we see either, that hitherto the location of the organs involved was erroneous; or that the fact of so many negative instances being possible indicates a hasty generalization, and possibly a different law; or further, that the method itself cannot in any great number of instances be relied upon as scientifically accurate. Most persons, and the majority of metaphysicians, willingly admit that women ordinarily possess more veneration than men, and the phrenologists, therefore, were quite justified in seeking for its appropriate locality. This, it is well known, was settled by them to be about the middle of the coronal surface, at the bregma or fontanel of the anatomists, the organ being "generally larger in the female head than in the male," to use Combe's own words.† "This," says Sir W. Hamilton, "I found to be the very reverse of truth, by a comparative average of nearly two hundred skulls of either sex. In man, the female encephalon is considerably smaller than that of the male, and in shape the crania of the sexes are different. The female skull is longer, it is nearly as broad, but it is much lower than the male. This is only one of several curious sexual differences of the head."‡ We may add, that the individual in whom Gall was first able to discover the organ, was his own brother, who was always engaged in praying or saying mass, and who eventually became a hermit and then a monk. The next instance is even more marked. No phrenologist, to our knowledge, has ever yet examined the head of a murderer without discovering the organ of destructiveness, and its cognates, to be very large, or over the healthy average; or, at any rate, has refrained from notifying it if discovered, lest, perhaps, so awkward a fact should be a sad blow to the very credibility of his science in the eyes of the vulgar. And yet so large a number of negative cases as are presently to be mentioned, reveal the very hastiness of the generalization which has established its present position and importance, whilst it at the same time enables us to discern how far the possession of certain facts by the observer's mind so colours his judgment as to detract from the scientific worth of his observations.

* *Study of Character*, p. 58.
† *System of Phrenology*, i. p. 346.
‡ *Lectures*, vol. i. Appendix III. p. 411.

A comparison of the crania of murderers preserved in the Anatomical Museum of the University of Edinburgh, with nearly two hundred ordinary skulls, taken at hazard, elicited the fact that these criminals "exhibited a development of the phrenological organs of destructiveness and other evil propensities *smaller*, and a development of the higher moral and intellectual qualities *larger*, than the average. Nay, more, the same result was obtained when the murderers' skulls were compared, not merely with a common average, but with the individual crania of Robert Bruce, George Buchanan, and Dr. David Gregory."*

Whether such facts as these affect phrenology as a whole or not, and they are not singular but simply best authenticated cases, they disclose a low standard of what constitutes evidence, and a complete absence of legitimate verification, according to modern canons. No one is careful to admit thus much more fully than Mr. Bain, even when disposed to think that such a procedure as the latter is by no means impossible. In his boldness upon this point, he declares that the doctrine that the brain, as a whole, is the organ of the mind, is demonstrated, but not *over*-demonstrated, and that very little less evidence than we have would suffice—a fact which is in reality more than borne out by the negative researches of Mr. G. H. Lewes and others. "Yet," he exclaims, "how very inferior the proofs of any one of the phrenological subdivisions of the brain being connected with a special faculty!" Reference is then made to the visible improvement in modern scientific evidence, and he continues:

"What would have been held good evidence at the time when Gall commenced his inquiries would not be counted so now, even in matters of natural history and natural philosophy. The inductive logic of John Stuart Mill has made the principles of experimental proof accessible to every student, and if we will but look at his chapter on 'Co-existences Independent of Causation,'† we shall find a clear account of the exact logical position of the phrenological affirmations. He points out that such propositions demand uniformity without a break, in order to establish them in their generality. There must not be one single real exception, otherwise the rule is as completely void as if there were not one instance in its favour. Consequently, every instance that seems to contradict the general affirmation must be met and shown to be only an apparent exception. . . . . But has this been done with any one of the phrenological organs?"‡ Anything like an experimental verification of the

* Ibid. † *Logic*, Book III. chap. 22.

‡ *Study of Character*, pp. 59, 60.

phrenological organs, however, is not possible artificially, so that in such a case, according to Mr. J. S. Mill, the only method whereby even approximate generalizations can be obtained upon matters affecting both individual character and social history, is the deductive method. Indeed, the whole of the same writer's remarks upon the chemical method in social science, with simply an alteration of terms, may be applied to phrenology, where, as we have said, artificial experimental verification is not possible, and the difficulty of ascertaining and noting the complete facts of each case, and determining the effect of latent and composite causes, are equally marked. And further, as confirming the general line of our criticism thus far, we append a portion of his valuable chapter in the "Logic of the Moral Sciences" on the "Laws of Mind."

"It must by no means be forgotten that the laws of mind may be derivative laws resulting from the laws of animal life, and that their truth, therefore, may ultimately depend on physical conditions; and the influence of physiological states or physiological changes in altering or counteracting the mental successions is one of the most important departments of psychological study; but, on the other hand, to reject the source of psychological analysis, and construct the theory of the mind solely on such data as physiology at present affords, seems to me as great an error in principle, and an even more serious one in practice. Imperfect as is the science of mind, I do not scruple to affirm that it is in a considerably more advanced state than the portion of physiology which corresponds to it, and to discard the former for the latter appears to me an infringement of the true canons of inductive philosophy, which must produce, and which does produce, erroneous conclusions in some very important departments of the science of human nature. . . . . The physiology, however, of the brain and nervous system is in a state of such rapid advance, and is continually bringing forth such new and interesting results, that if there be really a connexion between mental peculiarities and any varieties cognizable by our senses in the structure of the cerebral and nervous apparatus, the nature of that connexion is now in a fair way of being found out. The latest discoveries in cerebral physiology appear to have proved that any such connexion which may exist is of a radically different character from that contended for by Gall and his followers, and that whatever hereafter may be found to be true, phrenology at least is untenable."*

But we are here met by certain supposed obstinate facts on

---

* *System of Logic*, vol. ii. pp. 431, 439.

the part of phrenology as a science of mind and character, and especially the latter—namely, the affirmation of its followers, that, in spite of the confusions, deficiencies, and errors we may have noticed, phrenology has given, and continues to give, accurate and proved indications of individual character, on a scale which cannot be questioned. Such facts, however, are neither very obstinate nor very conclusive. The peculiar character of phrenological studies is, that it perpetually puts the student in an attitude of observation, no matter how carefully he may make his observations and how rigorously he may advance to his conclusions, and so far it must have some effect. The repeated attempt to sum up in a dashing off-hand way the chief features in the character of every acquaintance and every new individual he may meet, very often without any examination of organs at all, save those commonly exposed to the eye and capable of being measured by it, cannot in the end but result in a certain kind of skill, which does not become science because it may chance to be successful. Physiognomy, unquestionably, renders immense assistance in an almost numberless and indefinite variety of ways; and the tones of the voice, the terms of expression, the manner of behaviour and carriage, the dress, and the impression of the entire external man, contribute so large a share of the remainder, that if it could possibly be subtracted from the general estimate, would leave but a very dubious residuum indeed as the strict result of cranial manipulation. The case of professional phrenologists, who do actually and invariably tabulate the cranial indications, presents little more logical obstinacy. The estimation of character has become with them a profession, in which it is difficult to say whether scientific or rule-of-thumb proficiency is the predominating power, and the whole of their mental activity is so absorbed by it that it were next to impossible or miraculous that they should not in the majority of instances arrive at a number of tolerably accurate conclusions. Besides, there are many forms of character which become less occult and subtile in proportion to their more vigorous development; and here, as may be expected, their general success is greatest, as would be that of any intelligent, non-professional, and immethodical observer. The whole body and form of a man, as we have said, is an expression of himself in some form or other more or less difficult to be got at, of which every act and utterance, could we but see it in its true line of relation, is full of interpretative power; and it is the former fact which lies at the basis of all those immediate and first-sight impressions which are often subsequently so difficult to overcome, and upon which so large a portion of our fellow-men are content to lean in their social intercourses. Moreover, professional craniologists seldom trust themselves to the exact limits their science prescribes, and the muscular development, the nervous susceptibility, and the general health, are all determined by purely extraneous aids, or by direct questionings. Indeed, so far from evolving the general character by an accurate comparison of organs and their averages, this is only relied upon as one of many sources of knowledge. For we have only to consider for a moment the immense arithmetical difficulty incurred by a comparison of thirty-five organs in different degrees of development, with a standard for each one which can never be fixed as a rule having unexceptional authority, because a perfect type of a completely developed human brain and corresponding cranial indications is out of the question—to conceive the impossibility of the general result being the product of a strictly exclusive, scientific, and therefore demonstrable method. If the deficiencies, averages, and excesses have to be put over against each other, and reduced into intelligibility by an algebraical process, the result would be a whole composed of so many balances, modifications, checks, and counter-checks, as, if rigorously pursued, would issue in a statement more dubious and vague than ever uttered by an ancient oracle. Something like this, we believe, is nevertheless attempted by the better class of professional manipulators, who now and then discover to benighted individuals what, had they been wiser, they had needed no one to show them. We can only say this of the better class; for unfortunately, as even in his scientific enthusiasm for phrenology M. Comte felt constrained to prophecy, "from its vicious isolation, it tends to sink to the level of the most superficial and ill-prepared minds, which will make it the groundwork of a gross and mischievous quackery, if the true scientific inquirers do not take it out of their hands."* This does not now seem at all likely amongst ourselves, where the more advanced scientific minds are gradually slipping away from it, even when they have formerly clung to it as a sort of specific for what they regarded as brain-bewildering metaphysics, and the majority of the itinerating professors either are, or wish to be thought, Americans.

We are very far, therefore, from wishing to deny that with such important additions as the cleverest manipulator would hardly like to have altogether shut off from his use, phrenology has and does afford to some men a general but very loose insight to character. But this marks the very limit of our affirmative statement. That it is scientific, or can possibly be made so, without so far trenching upon other departments and borrowing

---

* *Positive Philosophy*, vol. i. p. 479.

from them upon every hand, as to lose its boasted distinctness as a science, and run counter to the precise path shaped out for it by the arrogance of its earliest champions, is quite another question, upon which our opinion is by this pretty plainly bodied forth. Good guessing is better than nothing, even when it is not what it pretends to be. We admit that as an estimate of individual traits, with powerful even when it may be half-unconscious helps, it is very often successful in the hands of the expert, but we have not yet considered how often it is unsuccessful. This is entirely a new aspect, but one by no means to be discarded, although not an easy one to satisfactorily establish. The handwriting is shown by Dr. Laycock* to be so far modified by conditions of the nervous system as to present some common characteristics of temperament and perhaps habits, and degenerations of the nervous system are seen to develope themselves in certain definite types of countenance and single abnormal features, but the deduction of further and preciser particularities would immediately lead from physiology into quackology. This expresses precisely the limits of phrenological truth. The facts it yields are so small, and subject to so many variations from unknown and composite causes, that anything like a full and minute mapping of the mind is impossible. To one good guess there are many failures. On the one side we may place a very loose and stereotyped statement of tendencies and facts which might suit any one, and would be sure to have some points of truth in it, and on the other a large and decided class of great and serious errors which would be more publicly made known did they not too often come in the pleasing form of a flattery which smooths over many inequalities, covers many genuine puzzles, and has the effect of working on behalf of the very science which has thus cunningly confessed itself foiled. We have been often amused at these rhetorical flourishes, and on comparing them with similar documents in which the character had been read from the handwriting, have been curiously struck with the same indefinite profound vacuity and complimentary terms. Every one, indeed, is usually astonished at himself, which, by the way, is perfectly unnecessary, for phrenology, as a science or prophecy of genius, is about as penetrative as palmistry and as barren as astrology. If only those disconsolate men and women would but honestly come forward who had been told that they had this, or that, or the other grand quality which as yet they had never been able to realize to themselves, and make a clean confession of everything, it would then be seen how far phrenology can be trusted as a science, and

---

* *Medical Times and Gazette,* Feb. 15th and 22nd, 1862.

how far the simple may be easily beguiled by a judicious admixture of learning and flattery. Nay, we can even venture to state that if the head of a murderer should by chance have been examined a couple of years before the fact that stamped him as one, it is a very fair question of doubt as to whether his evil propensities would have come out in the examination at all, so little distinction do they wish to draw between character before the act and character after the act. But only let the examination take place when the fact is fully known and consummated, so to speak, and the result invariably confirms, or is made to confirm, a strict organology. A clipping from the *Liverpool Mercury* of September last gives us a still further insight into these after-wisdoms and *à posteriori* processes:—

"The formation of the head of Taylor (whose name will be familiar to most of our readers) shows that his brain was malformed, and that his irrational conduct was in strict keeping with the type of his brain. The absolute volume of the brain was about the average, and from the nature of his temperament and scrupulous habit, the brain would act with great and protracted intensity. As regards the formation of his head, the ear is placed at an angle of 40 degrees in relation to the eyebrow. The vertical depth of the base of the brain from the opening of the ear to a horizontal line drawn from the centre of the ossification of the frontal bone is $3\frac{8}{10}$ in.; the width of the head over the ears is $6\frac{6}{10}$ in.; the vertical depth of the coronal, or region of the moral instruments of the mind, is $6\frac{6}{10}$ in. These measurements show that Taylor had a basilar brain, and that all the propensities we possess in common with the lower animals were in great excess over the moral feelings. The relative size of the phrenological organs, as indicated by the formation of the head, were as follows:—Amativeness: large. Philoprogenitiveness: rather large. Inhabitiveness: moderate. Concentrativeness: rather large. Adhesiveness: rather large. Marriage: full. Combativeness: large. Destructiveness: very large. Preservativeness: rather large. Alimentativeness: rather large. Acquisitiveness: large. Secretiveness: rather large. Cautiousness: rather small. Love of Approbation: large. Self-esteem: very large. Benevolence: rather small. Veneration: moderate. Firmness: large. Consciousness: small. Imitation: large. Marvellousness: average. Humorousness: full. Ideality: moderate. Sublimity: ditto. Individuality: ditto. Form: rather large. Size: rather large. Weight: rather large. Colour: average. Order: average. Number: rather large. Time: rather large. Constructiveness: rather large. Language: rather large. Comparison: rather large. Causality: rather small. It will be seen that the size of many organs in this development is large and powerful. Large amativeness is quite in keeping with his sensual course of life. The great size of destructiveness, combativeness, self-esteem, and firmness, with only small conscientiousness, and rather small benevolence, rendered him a most dangerous and formidable character, as they would give an implacable feeling of revenge, and he could take away the life of anything that stood in his way without the least compunction before or after the deed. Indeed, such a man is void of moral sense, and his excessive self-esteem inflated him with the idea of his own self-importance. Hence, whatever he did was right in his own estimation, and he would have deemed it impertinence in any one to question his judgment. Such a man would be a proud, conceited, vain boaster, a reckless speculator and gambler, and no one could have anything to do with him without being defrauded. Cautiousness and causality are low—hence his want of circumspection and prudence, and inability to trace out causation. In fact, the head is a low, miserable type, that could take pleasure in seeking the path of evil."

Probably such a complete phrenological success is rare in the annals of crime. At the same time few estimates of character contain so many inaccuracies, vague terms, general loosenesses, or so carefully play in the direction the facts of the man's life warranted. Now just let us try for a moment if no other combination is possible, by a similar system of tendency, spur, and check. Amativeness, *plus* philoprogenitiveness, marriage, inhabitiveness, adhesiveness, preservativeness, acquisitiveness, and self-esteem, would make a loving father, a home-keeping and worthy husband, and a careful and industrious man. Add combativeness, and we have a counteraction that might possibly affect the temper, and with destructiveness, a still further one that might indicate fits of strong and almost ungovernable passion. Again, we are met by a contra-stimulation in humorousness, love of approbation, and order. Cautiousness being small may show a bold disposition, but preservativeness and eventuality, again, being larger than causality, and time larger still, may supply somewhat of the deficiencies in this respect. Veneration, ideality, and sublimity being moderate, are to be set over against the smallness of benevolence and conscientiousness. Concentrativeness, firmness, comparison, and individuality, produce another complexity, whilst number, time, and language being rather large, stimulated by imitation, form, and self-esteem, all larger, the last on a par with destructiveness, would surely issue in something remarkable. Further, note that marvellousness is only an average, and even when backed by strong destructive and other selfish qualities, would scarcely lead to a love of distinction or notoriety for its own sake. Whether these combinations, aided by a brain acting with "great and protracted intensity," make upon the whole "a low, miserable type," is by no means clearly established even with the advantages afforded by antecedent facts. The man might equally have been a reformer, an orator, an actor, an engineer, a painter, a musician, or a wit; and so far from destructiveness necessarily being the moving force, selfishness, backed by any other qualities out of the category called large and rather large, were equally possible *à priori.* The estimate is nothing but a mere patchwork; and a judicious pairing-off of organs, had the man been singular in his lifetime in any of the other spheres indicated above, would have resulted in a similar complete triumph of the phrenological scheme. It is not in the mere tabulating of the faculties, but in their mysterious chemistry, that the real problem lies; and when facts have already determined this, it can be no difficult matter to make them correspond in the most seemingly irrefragable manner.

The prettiness of phrenological theory is indeed very captivating. To square-up a man according to his type is a very easy and convenient way of avoiding all the puzzles of human events, circumstances, and character. Organization is an apt tantalizing necessity which destroys duty, if carried out into detail, and satisfactorily annihilates all problematic moral distinctions. That a man is what he is, solely because he has not sufficient contrariness to be what he possibly might have been if he had not been what he was, is certainly an interesting form of shelving all chimeras whatever. This formula, we know, will be at once and readily denied. But in the case of Taylor, one cannot help asking, Who made destructiveness a ruling power? Did his tendency, or his circumstances, or both? The mysterious element of will is so carefully weeded out, that we cannot detect the least flash of its operation in this triumphant analysis. These are old questions, we know, and we would only wish them to have appositeness in reference to the case before us, since a consideration of them would lead us, and perhaps unfairly, beyond the limits of our present inquiry, and yet it was impossible not to touch upon them as showing what phrenology is really doing around us in the way of blind guesswork, and how much there is really to be advanced that deprives even facts of their supposed logic and obstinacy.

As an ultimate analysis of mind, phrenology is seen not to stand the test of present ideas and methods. But what shall we say in the light of the future? Where is the man, the social community, or the scientific nationality, that has caught up the light before us by virtue of phrenological culture?

Where are the newer types, the bolder forms, the ideal approximations? Surely, we should look for a phrenological phalanstery, and a Pepysian diarist? But let us remove the test into an abstract region. We may, or we may not, be suspicious of scientific finalities; but what shall we say of endeavours to reduce the fluctuating elements of mind into a solid and methodic form, where there is no ambiguous territory, no areas for extension, and no possibility allowed for in the up-heaving of new strata? To say that the map of London will always be the same as it is now, is something like proclaiming a modern phrenological chart which embraces the whole kingdom of the mind in all its future possibilities. There is, indeed, strictly speaking, hardly anything like a future in the calculations of this science. That human character is susceptible of improvement, is a corollary somehow attached to its supposed revelations, but how and where this improvement is to be effected is by no means clear, excepting that portions of the brain are to acquire an ascendancy over others. The contest of coming centuries is to be simply one of possession between two or three elements, all of which are already circumscribed by impassable limits. Hood's lines are the best description of the issue we have yet seen—

> "Knobby rivals,
> Tugging together like sheer devils,
> Till one gets master, good, or sinister,
> *And comes in like a new prime minister.*"

As for expansion, or fresh generative force, there is no room for it, and the impossibility is assumed without even so much as a hint, a reason, or a law. Men are to go on becoming more clearly only what they have been before more confusedly. The future brain is a compound exact and unyielding. In some inexplicable way the race shall gain new ideas, arts, sciences, and pursuits, without the possibility of a strictly novel type of brain, or the least change in the general arrangement of its faculties. Even the help the past might have afforded them in this light is discarded. For instance, memory among the ancients was vaster, richer, more unyielding than it is now, from the want of facilities possessed by the moderns; and yet this fact has no force, and a bust of Homer is mapped out precisely like a modern one, and is indeed made to yield its evidence in favour of the modern arrangement. Poetry was woven into almost every primitive science and study, and yet ancient busts are held to yield no other evidences of it except under ideality, comparison, and imitation. Wonder and veneration were mighty attributes, permeating and colouring the whole primitive life; and yet their modern organs are made to correspond without any regard to this whatever in their typical standard of organology. The riddle of the future, nevertheless, grows plainer by these retrospections. If education can change and is to change the phrenological aspects, how has it changed these? Are memory, ideality, wonder, and veneration softened down in the modern type? Have others arisen to establish a newer balance? If it has not changed them heretofore, where is the evidence that it ever will? Herein, to our thinking, lies one of the gravest errors in the whole of phrenological science. Collateral science may arrange for us certain definite types of brain characteristic of different portions of the earth's surface; but it attempts to do little more. It is here that phrenology should step in, for it opens an entirely new department of historical verification. Whether the head of Hiocien-Thsdug, the Buddhist pilgrim, differs from that of a modern theologian and philosopher of the same race and district, may be a difficult question to determine in the absence of any correct representation such as photography could give, but it is one that must be answered in some form or other before phrenology can have a historico-philosophical ground, and then a clearly-defined future. Pictures are inadmissible for this purpose, because perfection of detail cannot be expected in even the most masterly portraits, although phrenology has loosely pressed them in as evidence for modern positions, and only busts are sufficiently accurate for the purpose. But to come to periods more easily measurable. Two or three centuries of a critical character in any national European history, ought to exhibit some deviations in the way of general organology appreciable by the astute erudite phrenologist. Take, for instance, from our own nation a poet, a historian, a man of science, and a statesman from the fifteenth century, and compare them in detail with the busts or skulls (if they can be had) of similar eminent men living close upon the confluence of the last and the present century. Where do the differences lie? What group of faculties have gained or lost? If such an examination, honestly conducted, yield no appreciable results, then we cannot expect future ones, and the science has no tap-roots, and cannot be expected to have either very many branches or very much fruit.

Humanity, indeed, is more singularly unlike the vast tomes of German memoirs of which Carlyle complains so characteristically, than phrenological enthusiasts would have us believe: it wants something more than an index, especially one that is to be fixed and unyielding, for it is as yet incomplete, struggling, and progressive. As men ripen in thought, and the universe opens around them, they grow suspicious of these finalities that are to rid them of the trouble and the blessing of patient thinking, exact comparison, and cumulative truth. The attempts of centuries justify themselves even in their seeming failures, and the science of mind cannot afford to discard old methods for new ones that begin by promising to banish all perplexity, and end by making it only more profound.

---

### Art. VIII.—AMERICAN ASYLUMS FOR THE INSANE.*

